# Prevalence of Multiple Chemical Sensitivity in Canada Between 2000 and 2020

**DOI:** 10.3390/ijerph23020236

**Published:** 2026-02-13

**Authors:** Stephanie Robins, John Molot, Rohini Peris

**Affiliations:** 1 Association pour la Santé Environnementale du Québec-Environmental Health Association of Québec (ASEQ-EHAQ), Saint-Sauveur, QC J0R 1R1, Canada; stephanie.robins@uqo.ca (S.R.); jmolot@rogers.com (J.M.); 2 Faculty of Medicine, University of Ottawa, Ottawa, ON K1H 8M5, Canada

**Keywords:** multiple chemical sensitivity, MCS, Canada, prevalence, healthcare accessibility

## Abstract

**Highlights:**

**Public health relevance—How does this work relate to a public health issue?**

- Population-level data over two decades indicate that multiple chemical sensitivity (MCS) affects a growing share of the Canadian population, raising questions about population health burden.
- Marked variation in MCS prevalence by province, age, and sex points to differences in environmental exposure and healthcare system recognition that are relevant to public health surveillance.

**Public health significance—Why is this work of significance to public health?**

- This study synthesizes two decades of national survey data to present a clear, population-level picture of healthcare-professional-diagnosed multiple chemical sensitivity (MCS) in Canada.
- By documenting trends and demographic patterns over two decades, the findings contribute baseline evidence that is needed to interpret changes in MCS prevalence within and across populations.

**Public health implications—What are the key implications or messages for practitioners, policy makers and/or researchers in public health?**

- Prevalence estimates make MCS visible at a public health level and provide policy makers with clear, understandable measures of how many people are affected.
- Using population surveys allows prevalence estimates to be compared across regions, countries, and health systems, supporting planning, monitoring, and future research.

**Abstract:**

Multiple chemical sensitivity (MCS) describes an acquired condition characterized by recurrent, non-specific symptoms attributed to previously tolerated chemical exposure. Although Canada collects national data on MCS through population health surveys, the condition remains poorly understood and under-studied. This study analyzes data from the Canadian Community Health Survey (2000–2020) to examine trends in MCS prevalence across demographic characteristics, including province of residence, sex, and age. Descriptive analyses were used to assess temporal changes and differences between regions and population subgroups. Between 2000 and 2020, the proportion of Canadians reporting an MCS diagnosis increased from 1.9% to 3.5%. Prevalence varied geographically, with higher rates consistently observed in the Atlantic provinces compared with the Prairie provinces. MCS prevalence increased with age in both sexes; however, rates were higher among young males than females, with this pattern reversing in mid-life as prevalence became higher among females. These findings provide a national overview of MCS prevalence over two decades and offer relevant information for public health authorities, healthcare professionals, and researchers. The observed prevalence aligns with international estimates, underscoring MCS as an emerging public health concern in Canada.

## 1. Introduction

Multiple chemical sensitivity (MCS) is an acquired chronic condition characterized by adverse health effects linked to an exposure, or repeated exposure, to chemicals normally tolerated by the general population [1]. People with MCS may react to inhaled contaminants, such as mould, fragrances, exhaust, cigarette smoke, and poor-quality indoor or outdoor air; ingested products, including medications, food additives, or pesticides; and topical exposure, such as household cleaning products, bedding washed with fragranced laundry detergents, and personal care products [2,3]. Symptoms affect multiple body systems, and often include headaches, dizziness, heightened sense of smell, upper respiratory discomfort, chest and throat pain, irregular heartbeats, joint pain, skin irritation, memory difficulties, fatigue, confusion, and trouble concentrating [4,5]. Eliminating triggering exposure often leads to a reduction in symptoms [6]; however, the long-term consequences of MCS can be significant, negatively affecting physical and mental health as well as social, occupational, and daily life [7,8].

A substantial body of scientific literature describes the potential mechanisms underlying MCS [9]; however, widely accepted clinical biomarkers do not exist. A clinical diagnosis of MCS is based on six consensus-based criteria: “(1) the symptoms are reproducible with [repeated] chemical exposure; (2) the condition is chronic; (3) low levels of exposure [lower than previously or commonly tolerated] result in manifestation of the syndrome; (4) the symptoms improve or resolve when the incitants are removed; (5) responses occur to multiple chemically unrelated substances; and (6) symptoms involve multiple organ systems” [10]. To assist in diagnosing MCS, validated screening questionnaires have been developed. The most widely used and reported of these is the Quick Environmental Exposure and Sensitivity Inventory (QEESI) which assesses someone’s chemical exposure, the related symptoms and the impact on their life [11]. Otherwise, MCS is often self-diagnosed (with or without the terminology used in the literature), as people note patterns of symptoms they experience from exposure in their environment.

International classification of MCS remains varied. MCS is a recognized disability in Canada [12]. To provide a diagnostic code, countries such as Germany and Spain [13] use the International Classification of Diseases 10th revision (ICD-10) code 78.4, which broadly covers “allergic or hypersensitivity reactions, unspecified” [14]. Denmark uses DR688A1 for “symptoms related to chemicals and scents” a subheading to the ICD-10 code R68.8 [15]. Japan recognizes MCS under the ICD-10 code T65.9, which refers to the “toxic effect of an unspecified substance.” MCS is associated with sick house syndrome in Japan, as the conditions are considered to have similar symptoms and disease mechanisms, guiding diagnosis and treatment approaches [16]. Despite this growing global recognition, a dedicated ICD code for MCS has yet to be established by the World Health Organization.

Accordingly, the prevalence of MCS varies significantly across studies, as surveys include both self-reported cases and those diagnosed by healthcare professionals who may use different diagnostic criteria. Overall, MCS is more common in women, and due to its chronic nature, it tends to be most prevalent among older adults. Self-reported prevalence is typically higher than in physician-diagnosed cases, and estimates based on clinical diagnosis may further vary depending on the measurement used or the knowledge of the medical professional. When using the QEESI screening tool, studies have reported prevalences of 7.5% in Japan, 8.2% in Denmark, 10.3% in Spain, and between 12.8% (2018) and 20.6% (2023) in the USA [17,18,19,20,21]. When using a screening question that asks if the person has ever received a diagnosis of MCS from a healthcare professional, rates vary from 2.0% in Denmark, to 1.0% (in 2008) and 6.5% (in 2018) in Australia [22,23,24]. Canadian studies of MCS have relied on data from the Canadian Community Health Survey (CCHS), which captures health data from a representative sample of the population and levels of MCS as diagnosed by a healthcare professional [25]. The Women’s College Hospital of Ontario reviewed the CCHS data on MCS, reporting a mean prevalence of 2.5% for adults in the province of Ontario in 2005 [26]. Another study examining MCS and mental health outcomes found a prevalence of 2.83% when using CCHS data from 2012 [8].

The existing studies are largely based on single surveys or isolated time points, often using different definitions and measurement tools. This limits the understanding of how MCS prevalence evolves over time within populations. To date, there are no published population-based studies that track MCS prevalence consistently over multiple survey cycles using the same instrument. By analyzing data collected between 2000 and 2020, this study provides a rare longitudinal population-level perspective on changes in healthcare-professional-diagnosed MCS across age, sex, and province, addressing a critical gap in the literature. To better understand the prevalence of MCS in Canada over time, this report examines healthcare-professional-diagnosed rates of MCS from the CCHS data across provinces, age groups, and sexes between 2000 and 2020.

## 2. Materials and Methods

### 2.1. Participants

The CCHS data used in this analysis included survey respondents aged 12 and above. The following groups are excluded from the CCHS: persons living on reserves and other Aboriginal settlements; full-time members of the Canadian Armed Forces; institutionalized persons and those living in the Quebec regions of Nunavik and Terres-Cries-de-la-Baie-James.

### 2.2. Survey and Data Collection

Statistics Canada conducts the CCHS, a nationally representative cross-sectional survey that uses a multistage stratified cluster sampling design [25]. The survey collects information on health status, healthcare utilization and living conditions. For the study period (2000–2020), interviews were administered by trained interviewers using computer-assisted telephone or in-person methods with standardized questionnaires and centralized quality control procedures.

Multiple chemical sensitivity (MCS) was identified using the question: “Do you suffer from multiple chemical sensitivities?” Respondents were instructed to answer “Yes” only if symptoms had persisted for at least six months and a diagnosis had been made by a healthcare professional. One eligible respondent per household was selected using probability-based methods. All analyses applied person-level survey weights to account for unequal selection probabilities and non-response, with variance estimation conducted using CCHS bootstrap replicate weights to calculate standard errors and 95% confidence intervals, following Statistics Canada’s analytic guidelines for the CCHS [27].

A survey cycle represents a full round of data collection and yields an independent, nationally representative dataset with dedicated design and bootstrap weights; some earlier cycles spanned two calendar years rather than a single calendar year. Survey cycles from 2021 onward were excluded because the CCHS underwent a redesign that introduced online self-administered data collection alongside interviewer follow-up, which may affect comparability with earlier cycles. Restricting analyses to pre–redesign cycles ensured methodological consistency.

### 2.3. Data Analysis

Descriptive statistics report the overall prevalence of MCS, as well as prevalence by province, age and sex. We used Microsoft Excel (Microsoft 365; Microsoft Corp., Redmond, WA, USA) for calculations and table creation. See Supplementary Materials for the complete dataset.

## 3. Results

### 3.1. Prevalence of MCS in Canada

**Figure 1:**
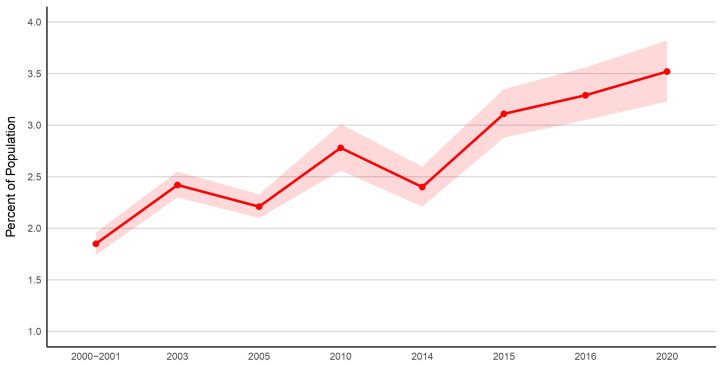
Percentage of people diagnosed with MCS in Canada 2000–2001 cycle to 2020. Note: Solid dots represent average percentage, and shaded areas represent 95% lower and upper confidence intervals.

In the 2000–2001 survey cycle, the reported prevalence of MCS in Canada was 1.8%, rising to 2.8% in 2010, and by 2020, 3.5% of Canadians (*n* = 1,130,801) had been diagnosed with MCS (Table 1). The overall increase in MCS is illustrated in Figure 1.

### 3.2. MCS Cohort Sex Differences

Of the 2020 MCS total cohort (*n* = 1,130,801), 27.9% (*n* = 315,149) were male, and 72.1% (*n* = 815,652) were female (Table 2).

### 3.3. Prevalence of MCS Across Provinces

Rates of MCS varied across provinces and over time (Table A1, see Appendix A). In the 2000–2001 survey cycle, Newfoundland reported the lowest prevalence of MCS at 1.1%, while Nova Scotia had the highest at 3.0%. Twenty years later, Alberta recorded the lowest rate at 2.4%, and Nova Scotia the highest at 6.8%. While all provinces have seen an increase in MCS prevalence, the largest increases overall were observed in Saskatchewan, Newfoundland and Labrador, Prince Edward Island, and New Brunswick.

### 3.4. Provincial Breakdown of MCS Cohort by Sex (2020)

**Figure 2:**
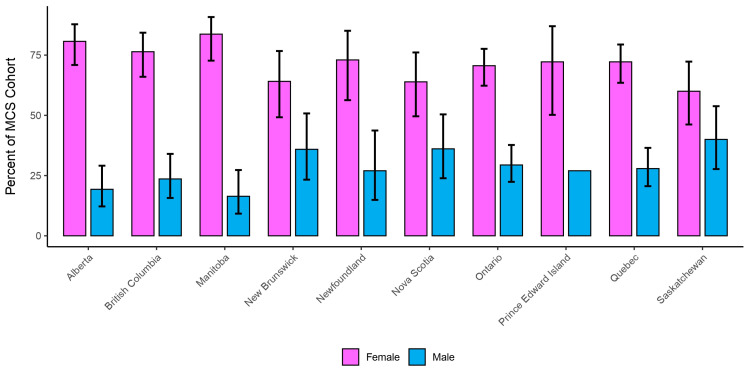
Proportion of males and females within MCS Cohort per province (2020). Note: Error bars represent 95% lower and upper confidence intervals, except for Prince Edward Island, where confidence intervals were suppressed due to insufficient reliability.

The ratio of MCS experienced by males and females differs by province, although females consistently outnumber males (see Figure 2). The 2020 survey cycle demonstrates differences between provinces, with Saskatchewan having the smallest gap between the sexes where males represent 40% of the cohort, and females 60%. Manitoba has the largest gap, with males reporting 16.5% and females 83.5%.

### 3.5. Distribution of MCS by Age and Sex

**Figure 3:**
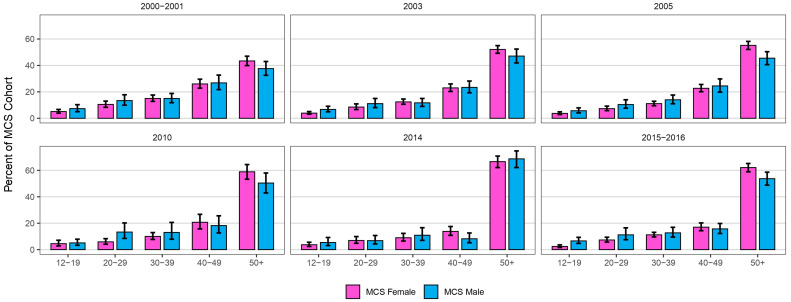
Distribution of MCS by age and sex in the Canada 2000–2001 cycle to 2016 cycle. Error bars represent 95% lower and upper confidence intervals. Note: Data on sex distributions were unavailable for the 2020 cycle.

The age range of those diagnosed spanned from 12 to 91 years. While females are diagnosed almost three-fold more than males, for all survey cycles, the prevalence of MCS increases with age for both males and females. In the 12–19, 20–29, and 30–39 age groups, males had higher rates of MCS diagnosis (7.2%, 13.5% and 26.8%, respectively) compared to females (5.1%, 10.4% and 26.0%) (see Figure 3). However, in the 40–49 and 50+ age groups, MCS was slightly less common in males (14.9% and 37.6%) than in females (15.0% and 43.3%).

## 4. Discussion

This report documents an increasing prevalence of MCS among Canadians aged 12 and older. Between 2000 and 2020, prevalence rose from 1.9% to 3.5% of the population. Similar upward trends have been observed in Australia [22,23] and the United States [20,21] where MCS prevalence was assessed using the same survey at two different time points.

As the second largest country in the world by land area, Canada spans highly diverse environmental and healthcare contexts across regions. The Territories and Atlantic provinces generally reported higher MCS prevalence than central and western provinces. In the 2020 survey cycle, the Atlantic provinces—Newfoundland and Labrador, Prince Edward Island, Nova Scotia, and New Brunswick—reported rates ranging from 4.6% to 6.8%, well above the national average. These geographic differences may reflect variation in environmental exposure, healthcare access, or diagnostic recognition across regions. An interactive Canadian prevalence map has been published elsewhere to visually contextualize provincial differences [28].

In 2020, 72.1% of respondents reporting MCS were female, compared to 27.9% who were male. This is consistent with population-based studies of MCS from Denmark and Japan, where 77.5% and 67.1% of respondents were female, respectively [17,24]. In Canada’s younger age group, however, males reported higher prevalence than females, whereas females reported higher rates later in life (see Figure 3). As the CCHS does not capture behavioural or clinical mechanisms underlying sex- and age-specific differences, we interpret these patterns cautiously. Potential explanations have been discussed elsewhere, including social science analyses of gender and illness experience in MCS [29]; epidemiological patterns by age and sex in general populations [30]; and biological responses to the environment via receptor sensitization [31].

MCS prevalence increased with age for both sexes, supporting the view that MCS is a chronic condition affecting a growing share of the Canadian population. This trend occurs alongside broader demographic changes, as Canada’s population continues to age; the proportion of adults aged 65 years and older has increased from approximately 14% in 2010 to nearly one in five Canadians (19.5%) in 2025 [32]. As older adults generally require greater healthcare support, rising MCS prevalence within an ageing population may translate into increased service needs and system-level pressures. Furthermore, studies reveal that those affected by MCS require complex, integrated, person-centred care [33]. In the absence of established curative treatments, this expanding number of affected people represents a foreseeable and growing public health challenge.

Beyond confirming international trends, this study provides a uniquely Canadian contribution by leveraging two decades of nationally representative CCHS data to describe temporal trends, provincial variability, and age- and sex-specific patterns in MCS prevalence. These findings challenge the assumption that prevalence is uniform and suggests that contextual, environmental, and health system factors may influence symptom reporting or diagnosis. By documenting these population-level patterns within a consistent surveillance framework, this analysis establishes a national baseline that supports ongoing monitoring, public health planning and future research.

## 5. Conclusions

The current analysis adds to the growing literature by providing nationally representative estimates of MCS prevalence in Canada. From a public health perspective, these estimates make MCS visible at the population level and offer decision-makers clear measures of how many Canadians are affected. The use of repeated CCHS cycles enables comparisons across regions and over time, supporting surveillance, planning and future research. Together, these data establish a national baseline against which future cycles can monitor change over time and refine understanding of the evolving burden of MCS in Canada.

### Limitations

No inferential analyses were conducted; therefore, causal mechanisms cannot be inferred. Additional limitations include methodological changes across survey cycles. Statistics Canada removed the MCS question between 2017 and 2019 and reinstated it in 2020. For Nunavut, Yukon, and the Northwest Territories, 2015–2016 data were reported jointly rather than as separate annual estimates, limiting year-specific analyses; these values are presented for 2015 in Table 2. Future research using consistent inclusion of the MCS question across cycles, year-specific reporting in all regions, and analytic or longitudinal designs may help assess determinants of MCS and address questions that cannot be examined using descriptive data alone.

## Figures and Tables

**Table 1:** Prevalence of multiple chemical sensitivity in Canadians (2020).

Population	*N*	Percentage (Range)	95% Confidence Interval Low	95% Confidence Interval High
Canadian population	32,165,545			
Total MCS cohort	1,130,801	3.5 (2.4–6.8 ^a^)	3.2	3.8

Note: ^a^ describes the range (%) between the lowest reporting province of 2020 (Alberta) and the highest (Nova Scotia).

**Table 2:** MCS cohort descriptive statistics.

MCS Cohort	*N*	Percentage	95% Confidence Interval Low	95% Confidence Interval High
Total cohort	1,130,801			
Male (% of cohort)	315,149	27.9	24.1	32.0
Female (% of cohort)	815,652	72.1	68.0	75.9

## Data Availability

The datasets presented in this article are not readily available because the primary data were obtained from Statistics Canada and are subject to legal and confidentiality restrictions. Access to the Canadian Community Health Survey data is available through Statistics Canada Research Data Centres with the permission of Statistics Canada. A minimal analytic dataset sufficient to reproduce the analyses is included in the Supplementary Materials. Requests for further information should be directed to the corresponding author.

## Supplementary Materials

The following supporting information can be downloaded at: https://www.mdpi.com/article/10.3390/ijerph23020236/s1, Table S1. Percentage of people diagnosed with MCS from 2000 to 2020 in Canada; Table S2. Percentage of people diagnosed with MCS for survey cycle 2000–2020 in Canada and breakdown of MCS cohort by sex; Table S3. Prevalence of multiple chemical sensitivity (MCS) in Canada and by province (2000–2020); Table S4. Percentage of people diagnosed with MCS from 2000 to 2020 in Canada; Table S5. Distribution of males and females with MCS by province (2020); Table S6. Prevalence of MCS in Canada by age and sex (2000–2001 and 2015–2016); Table S7. Distribution of MCS by age and sex in Canada from the 2000–2001 cycle to 2016 cycle.

## Abbreviations

The following abbreviations are used in this manuscript:

MCS	Multiple chemical sensitivity
CCHS	Canadian Community Health Survey
QEESI	Quick Environmental Exposure and Sensitivity Inventory

## Appendix A

**Table A1:** Prevalence of multiple chemical sensitivity (MCS) in Canada by province (2000–2020).

Region	2000–2001 *						2003						2005						2010					
	*n*	95% Confidence Interval		%	95% Confidence Interval		*n*	95% Confidence Interval		%	95% Confidence Interval		*n*	95% Confidence Interval		%	95% Confidence Interval		*n*	95% Confidence Interval		%	95% Confidence Interval	
		*n* low	*n* high		% low	% high		*n* low	*n* high		% low	% high		*n* low	*n* high		% low	% high		*n* low	*n* high		% low	% high
Canada	475,972	446,458	505,486	1.8	1.7	2.0	643,251	610,462	676,039	2.4	2.3	2.6	598,682	567,359	630,005	2.2	2.1	2.3	800,562	736,027	865,097	2.8	2.6	3.0
Newfoundland	5224 ^E^	3406	7042	1.1 ^E^	0.8	1.6	9930	7029	12,832	2.2	1.6	2.9	10,663	7778	13,548	2.4	1.8	3.1	12,138 ^E^	7993	16,284	2.7 ^E^	1.9	3.8
Prince Edward Island	2246 ^E^	1552	2940	1.9 ^E^	1.4	2.6	2652	1917	3387	2.2	1.7	3.0	2854 ^E^	1809	3898	2.4 ^E^	1.7	3.5	2988 ^E^	1771	4205	2.4 ^E^	1.6	3.7
Nova Scotia	23,786	18,965	28,607	3	2.5	3.7	23,147	18,572	27,722	2.9	2.4	3.5	22,185	17,677	26,694	2.8	2.3	3.4	31,316 ^E^	21,534	41,099	3.9 ^E^	2.8	5.3
New Brunswick	11,057	8597	13,516	1.7	1.4	2.2	21,348	17,190	25,505	3.3	2.8	4.1	17,887	14,122	21,652	2.8	2.3	3.5	21,829 ^E^	15,357	28,300	3.4 ^E^	2.5	4.6
Quebec	166,459	148,546	184,372	2.7	2.4	3.0	182,723	160,733	204,712	2.9	2.5	3.2	171,088	153,951	188,224	2.6	2.4	2.9	178,192	147,329	209,055	2.6	2.2	3.1
Ontario	150,286	131,105	169,467	1.5	1.3	1.7	233,882	213,170	254,594	2.3	2.1	2.5	217,924	197,661	238,188	2.1	1.9	2.3	292,658	249,859	335,456	2.6	2.2	3.0
Manitoba	12,025	8854	15,196	1.3	1	1.7	17,360	12,514	22,206	1.9	1.4	2.5	15,346	11,600	19,092	1.6	1.3	2.1	31,782 ^E^	17,937	45,628	3.2 ^E^	2.1	5.0
Saskatchewan	11,581	8613	14,549	1.4	1.1	1.9	17,172	13,667	20,677	2.2	1.8	2.6	17,105	14,025	20,186	2.2	1.8	2.6	23,803	17,520	30,086	2.9	2.2	3.7
Alberta	41,042	33,303	48,782	1.7	1.4	2	43,470	35,609	51,331	1.7	1.4	2.0	49,050	38,228	59,871	1.8	1.5	2.3	85,525	63,259	107,791	2.8	2.1	3.6
British Columbia	51,209	43,604	58,815	1.5	1.3	1.7	90,278	79,714	100,841	2.6	2.3	2.9	72,598	62,198	82,997	2.0	1.8	2.3	115,916	91,030	140,802	3.0	2.4	3.7
Territories	1056 ^E^	676	1437	1.4 ^E^	1.0	2.0	1290 ^E^	855	1725	1.8 ^E^	1.3	2.5	1982 ^E^	952	3012	2.6 ^E^	1.5	4.3	4414 ^E^	2935	5893	5.4 ^E^	3.9	7.5
Region	2014						2015						2016						2020					
	*n*	95% Confidence Interval		%	95% Confidence Interval		*n*	95% Confidence Interval		%	95% Confidence Interval		*n*	95% Confidence Interval		%	95% Confidence Interval		*n*	95% Confidence Interval		%	95% Confidence Interval	
		*n* low	*n* high		% low	% high		*n* low	*n* high		% low	% high		*n* low	*n* high		% low	% high		*n* low	*n* high		% low	% high
Canada	722,630	664,393	780,867	2.4	2.2	2.6	940,516	869,187	1,011,844	3.1	2.9	3.4	1,008,393	929,801	1,086,984	3.3	3.0	3.6	1,130,801	1,035,883	1,225,720	3.5	3.2	3.8
Newfoundland	7592 ^E^	4840	10,345	1.7 ^E^	1.2	2.4	16,255 ^E^	10,694	21,815	3.6 ^E^	2.5	5.0	16,982 ^E^	10,901	23,064	3.7 ^E^	2.6	5.3	28,527 ^E^	17,936	39,117	6.2 ^E^	4.3	8.9
Prince Edward Island	445 ^E^	2706	6204	3.6 ^E^	2.4	5.2	2815 ^E^	1619	4011	2.2 ^E^	1.5	3.4	4215 ^E^	2750	5681	3.3 ^E^	2.4	4.7	6342 ^E^	3417	9266	4.6 ^E^	2.9	7.2
Nova Scotia	29,449	21,485	37,413	3.6	2.8	4.8	30,437	22,747	38,127	3.8	2.9	4.8	35,005	28,144	41,867	4.3	3.5	5.2	57,529	41,258	73,801	6.8	5.1	9.0
New Brunswick	25,003	19,124	30,881	3.9	3.1	4.9	31,714	23,867	39,562	4.9	3.8	6.3	22,006	16,695	27,317	3.4	2.7	4.4	41,336	29,565	53,107	6.2	4.6	8.2
Quebec	152,672	125,348	179,995	2.2	1.8	2.6	173,514	145,422	201,607	2.5	2.1	2.9	230,530	197,880	263,180	3.2	2.8	3.7	250,973	201,316	300,630	3.4	2.8	4.2
Ontario	247,278	215,472	279,085	2.1	1.9	2.4	403,837	349,569	458,104	3.4	3.0	3.9	404,906	342,128	467,683	3.4	2.9	4.0	418,986	355,207	482,765	3.3	2.8	3.9
Manitoba	25,138	18,445	31,832	2.5	1.9	3.2	30,336	22,931	37,741	3	2.3	3.8	30,363	21,915	38,811	2.9	2.2	3.9	38,293	27,611	48,976	3.5	2.7	4.6
Saskatchewan	25,210	19,084	31,336	2.8	2.2	3.6	31,661	22,778	40,544	3.5	2.7	4.7	27,983	20,300	35,666	3.1	2.4	4.1	37,735	27,617	47,853	4.1	3.1	5.3
Alberta	103,586	79,583	127,589	3.0	2.4	3.8	81,587	64,574	98,600	2.3	1.9	2.9	89,254	70,749	107,758	2.5	2.1	3.1	87,720	67,560	107,880	2.4	1.9	3.0
British Columbia	98,031	77,541	118,520	2.5	2	3.1	138,360	115,533	161,187	3.5	2.9	4.1	147,149	123,213	171,086	3.6	3.1	4.3	163,361	129,102	197,620	3.7	3.0	4.5
Territories	4215	3049	5380	4.5	3.4	5.9	3171	2316	4026	3.4	2.6	4.4	**						n/a					

Note: ^E^—interpret with caution due to CV threshold (data have a coefficient of variation between 15% and 35%). * Data for the years 2000 and 2001 are combined. ** Data for the Territories is from a combined 2015 and 2016 survey.

## References

[1] Miller C.S. (1996). Chemical sensitivity: Symptom, syndrome or mechanism for disease?. Toxicology.

[2] Steinemann A. (2016). Fragranced consumer products: Exposures and effects from emissions. Air Qual. Atmos. Health.

[3] Masri S., Miller C.S., Palmer R.F., Ashford N.A. (2021). Toxicant-induced loss of tolerance for chemicals, foods, and drugs: Assessing patterns of exposure behind a global phenomenon. Environ. Sci. Eur..

[4] Fares-Medina S., Díaz-Caro I., García-Montes R., Corral-Liria I., García-Gómez-Heras S. (2022). Multiple Chemical Sensitivity Syndrome: First symptoms and evolution of the clinical picture. Int. J. Environ. Res. Public Health.

[5] Del Casale A., Ferracuti S., Mosca A., Pomes L.M., Fiaschè F., Bonanni L., Borro M., Gentile G., Martelletti P., Simmaco M. (2020). Multiple Chemical Sensitivity Syndrome: A principal component analysis of symptoms. Int. J. Environ. Res. Public Health.

[6] Perales R.B., Palmer R.F., Rincon R., Viramontes J.N., Walker T., Jaén C.R., Miller C.S. (2022). Does improving indoor air quality lessen symptoms associated with chemical intolerance?. Prim. Health Care Res. Dev..

[7] Dantoft T.M., Andersson L., Nordin S., Skovbjerg S. (2015). Chemical intolerance. Curr. Rheumatol. Rev..

[8] Johnson D., Colman I. (2017). The association between multiple chemical sensitivity and mental illness. J. Psychosom. Res..

[9] Zucco G.M., Doty R.L. (2022). Multiple Chemical Sensitivity. Brain Sci..

[10] (1999). Multiple Chemical Sensitivity: A 1999 Consensus. Arch. Environ. Health.

[11] Miller C.S., Prihoda T.J. (1999). The Environmental Exposure and Sensitivity Inventory (EESI). Toxicol. Ind. Health.

[12] Canadian Human Rights Commission (2019). Environmental Sensitivity and Scent-Free Policies.

[13] Ministerio de Sanidad, Servicios Sociales e Igualdad (2018). Clasificación Internacional de Enfermedades.

[14] World Health Organization (2016). International Statistical Classification of Diseases and Related Health Problems.

[15] Elberling J., Bonde J.P.E., Vesterhauge S., Bang S., Linneberg A., Zachariae C., Johansen J.D., Blands J., Skovbjerg S. (2014). A new classification code is available in the Danish health-care classification system for patients with symptoms related to chemicals and scents. Ugeskr. Laeger.

[16] Ishibashi M., Tonori H., Miki T., Miyajima E., Kudo Y., Tsunoda M., Sakabe K., Aizawa Y. (2007). Classification of patients complaining of sick house syndrome and/or MCS. Tohoku J. Exp. Med..

[17] Azuma K., Uchiyama I., Katoh T., Ogata H., Arashidani K., Kunugita N. (2015). Prevalence and characteristics of chemical intolerance. Arch. Environ. Occup. Health.

[18] Skovbjerg S., Berg N.D., Elberling J., Christensen K.B. (2012). Evaluation of the Quick Environmental Exposure and Sensitivity Inventory in a Danish Population. J. Environ. Public Health.

[19] Pérez-Crespo J., Lobato-Cañón R., Solanes-Puchol Á. (2018). Multiple Chemical Sensitivity in chemical laboratory workers. Saf. Health Work.

[20] Miller C.S., Palmer R.F., Kattari D., Masri S., Ashford N.A., Rincon R., Perales R.B., Grimes C., Sundblad D.R. (2023). What initiates chemical intolerance? Findings from a large population-based survey of US adults. Environ. Sci. Eur..

[21] Steinemann A. (2018). National prevalence and effects of MCS. J. Occup. Environ. Med..

[22] Fitzgerald D. (2008). Studies on self-reported MCS in South Australia. Environ. Health.

[23] Steinemann A. (2018). Prevalence and effects of MCS in Australia. Prev. Med. Rep..

[24] Dantoft T.M., Nordin S., Andersson L., Petersen M.W., Skovbjerg S., Jørgensen T. (2021). Multiple chemical sensitivity described in the Danish general population: Cohort characteristics and the importance of screening for functional somatic syndrome comorbidity—The DanFunD study. PLoS ONE.

[25] Statistics Canada (2020). Canadian Community Health Survey (CCHS): Annual Component 2020.

[26] Marshall L., Bested A., Molot J., Kerr K., Bray R.I. (2011). Environmental Sensitivities—Multiple Chemical Sensitivities Status Report 2010.

[27] Statistics Canada (2024). Canadian Community Health Survey (CCHS) User Guide.

[28] Environmental Health Association of Québec (ASEQ-EHAQ) Prevalence of MCS in Canada, 2020. ASEQ-EHAQ. https://aseq-ehaq.ca/en/prevalence-of-mcs-in-canada-2020-2/.

[29] Nadeau G., Lippel K. (2014). From individual coping strategies to illness codification: The reflection of gender in social science research on multiple chemical sensitivities (MCS). Int. J. Equity Health.

[30] Lu X., Hojo S., Mizukoshi A., Katoh T. (2023). Prevalence and correlation of multiple chemical sensitivity and electromagnetic hypersensitivity with age, sex, and depression in the Japanese population: A retrospective study. BMC Public Health.

[31] Molot J., Sears M., Anisman H. (2023). Multiple chemical sensitivity: It’s time to catch up to the science. Neurosci. Biobehav. Rev..

[32] Statistics Canada (2025). Canada’s Population Estimates: Age and Gender, July 1, 2025.

[33] Damiani G., Alessandrini M., Caccamo D., Cormano A., Guzzi G., Mazzatenta A., Micarelli A., Migliore A., Piroli A., Bianca M. (2021). Italian Expert Consensus on Clinical and Therapeutic Management of Multiple Chemical Sensitivity (MCS). Int. J. Environ. Res. Public Health.

